# Assessment of Perspectives on Health Care System Efforts to Mitigate Perceived Risks Among Immigrants in the United States: A Qualitative Study

**DOI:** 10.1001/jamanetworkopen.2020.3028

**Published:** 2020-04-17

**Authors:** Altaf Saadi, Uriel Sanchez Molina, Andreé Franco-Vasquez, Moira Inkelas, Gery W. Ryan

**Affiliations:** 1 Department of Neurology, Harvard Medical School, Massachusetts General Hospital, Boston, Massachusetts; 2 Dominican University, River Forest, Illinois; 3 David Geffen School of Medicine, University of California, Los Angeles, Los Angeles; 4 Department of Health Policy and Management, Fielding School of Public Health, University of California, Los Angeles, Los Angeles; 5 Health Systems Science Department, Kaiser Permanente Bernard J. Tyson School of Medicine, Pasadena, California

## Abstract

**Importance:**

Increases in the enforcement of immigration policies, deportations, and rhetoric critical of immigration during and after the 2016 US presidential election have been associated with a decrease in health-seeking behaviors and an increase in adverse health outcomes among immigrants. Efforts to address the health care needs of immigrants after the 2016 presidential election have centered on individual-level patient-practitioner strategies or federal- and state-level policy changes. However, these approaches have not captured the role of health care systems and the range of health care facilities encompassed within them.

**Objective:**

To characterize policies and actions implemented by health care facilities to address immigration status–related stressors.

**Design, Setting, and Participants:**

This exploratory qualitative study involved semistructured interviews in a purposive sample of health care facilities across 5 states (California, Texas, New York, Florida, and Illinois) with the largest populations of individuals with undocumented immigration status. Data from media sources and informational interviews with local immigration advocacy leaders were used to identify health care facilities that had implemented welcoming policies and strategies. Stakeholders, including administrators, frontline clinicians involved in policy implementation, and senior executive leaders, were interviewed. Interviews were conducted between May 1 and August 9, 2018, and were recorded, transcribed, and coded using constant comparative analysis. Data analysis was performed from June 29, 2018, to February 5, 2019.

**Main Outcomes and Measures:**

Stakeholder perspectives on the range, scope and content of policies and actions implemented to address immigration-related stressors at health care facilities.

**Results:**

A total of 38 in-depth interviews were conducted spanning 25 health care facilities in 5 states; these facilities included 13 federally qualified health centers, 7 academic or private hospitals, and 5 public institutions. Interviewees described policies and actions that mitigated one or more of the following perceived risks: (1) risk of exposure to immigration enforcement personnel at or near facilities, (2) risk of immigration status–related information disclosure, (3) risk associated with patient-level stressors, (4) risk associated with practitioner-level stressors, and (5) coordination of risk mitigation. Most personnel at health care facilities emphasized that their policies and actions fit within a larger mission and history of addressing the social needs of diverse patients and mitigating risks for patients.

**Conclusions and Relevance:**

Health care facilities can implement both active and reactive measures to address perceived immigration-associated risks among patients and practitioners. Population health and immigration policies are at the forefront of current policy debates. An understanding of the ways in which health care facilities can serve to mitigate perceived risks among their patients and employees can be one step toward optimizing health care for immigrants.

## Key Points

**Question:**

How have health care facilities in 5 states with the largest populations of individuals with undocumented immigration status responded to enforcement of immigration policies after the 2016 US presidential election?

**Findings:**

In this qualitative study involving 38 interviews across 25 health care facilities, such facilities were found to have implemented institutional policies and actions to mitigate perceived risks among patients who are immigrants and health care practitioners. Patients and practitioners identified risks related to exposure to immigration enforcement personnel at or near facilities and of immigration status disclosure; these risks were associated with patient-level stressors, with practitioner-level stressors, and with coordination of risk mitigation.

**Meaning:**

This study suggests that understanding the ways in which health care facilities address risks to their patients and employees may help to optimize care for patients who are immigrants and health care practitioners.

## Teaser

This qualitative study explores the policies and actions implemented by US health care facilities after the 2016 presidential election in an effort to mitigate perceived risks among immigrants and health care practitioners.

## Introduction

Studies have suggested that increased enforcement of immigration policies, deportations, and rhetoric critical of immigration during and after the 2016 US presidential election have reduced the willingness of immigrants to access health and social services^1,2,3^ and have been associated with adverse health outcomes, such as decreases in birth weights and increases in mental health disorders.^4,5^ Similar consequences have been documented after the increased enforcement of state immigration policies^6,7,8^ and the occurrence of immigration enforcement actions in the workplace.^9,10^ The consequences may extend to the physical and mental health of US-born ethnic minority populations who are perceived to be immigrants.^11,12^ Children born in the United States in families with mixed immigration status may also experience consequences. For instance, 1 in 4 American children with Latino ancestry belong to families with mixed immigration status^13^; such families appear to have lower than expected rates of participation in federally funded health and social programs, such as Medicaid; the Special Supplemental Nutrition Program for Women, Infants, and Children; and the Supplemental Nutrition Assistance Program.^14,15^

As a consequence, some health care facilities, such as hospitals and clinics, are adopting policies and actions to welcome immigrants, address immigration status–related stressors, and mitigate fears associated with accessing health care services. These efforts are embedded within a larger social and political landscape that includes media reports of immigration enforcement actions at or near health care facilities^16^ and state or city sanctuary policies limiting the involvement of local law enforcement agencies with federal immigration enforcement agencies.^16^ The ways in which health care facilities are responding to the increased enforcement of immigration policies has been discussed in media and medical literature editorials but has not been empirically studied. This study offers a systematic multistate exploration of institutional policies and actions undertaken to mitigate perceived risks among patients who are immigrants and health care practitioners.

## Methods

### Study Design and Sample

This study used a 3-stage sampling design. First, we purposefully selected the 5 states (California, Texas, New York, Florida, and Illinois) with the largest populations of individuals with undocumented immigration status.^17^ Within each state, we used informational interviews with local immigration advocacy leaders to identify 38 health care facilities that had implemented welcoming policies and strategies. One of us (A.S.) identified these local leaders from community partnerships established in previous immigration status–related advocacy efforts. This snowball sampling procedure was supplemented by Google searches to identify news articles written about sanctuary clinics and hospitals within each state. We contacted stakeholders at each institution to recruit study participants. Stakeholders included administrators, frontline health care practitioners involved in policy implementation, and senior executive leaders. We performed the study using the Standards for Reporting Qualitative Research (SRQR) reporting guideline. All study procedures were approved by the institutional review board of the University of California, Los Angeles. All participants provided verbal informed consent.

### Data Collection

We developed a 45- to 60-minute semistructured interview guide based on input from the literature and community organizations.^18^ Drawing from the Consolidated Framework for Implementation Research,^19^ the interview protocol included questions about potential barriers and facilitators to the implementation of risk-reduction strategies (Box).

Box. **Interview Guide**

1. Tell me about the history of how you/your institution became involved in implementing these interventions?
2. What are the range of policies you have implemented at your institution? What have you considered implementing?
  - Hospital/administrative interventions
  - Provider-focused interventions
  - Patient-focused interventions inside and outside the hospital setting
  - Policies promoting the use of health care services
  - Policies outlining boundaries of interactions with immigration enforcement personnel
3. Can you tell me about how these policy interventions were decided on and implemented?
  - Leadership?
  - Champions? Teams?
  - Community or legal consultation?
  - Patient involvement?
  - Resources?
4. What was difficult about the process? What challenges or resistance did you face? What did you have to overcome? What could you not overcome? (Ask for each intervention.)
5. What would you do differently?
6. What would you recommend to other people?
7. Tell me about the plans for evaluation of these interventions? How will you measure “success”?
8. Tell me about the challenges you perceive to affect immigrant populations and how they have changed over the past 2 years. What kind of local or state measures or policies have influenced these challenges?
9. Can you speak to how this local/state policy context has affected your health care institution in particular?
10. Do you know other health care facilities that have implemented similar policies?

Two of us (A.S. and A.F.V.) conducted interviews between May 1 and August 9, 2018. We conducted most of the interviews in person, with the remainder conducted by telephone. Interviewees provided verbal informed consent for audio recording and transcription in all but one instance, in which extensive notes were taken instead. Most of the interview refusal reasons were nonspecific because the individuals did not respond. Two individuals who declined to participate implied that the bureaucratic requirements at their institutions were too difficult to overcome to allow for an interview. Interviews continued until we reached thematic saturation (ie, until no new thematic information could be obtained).^20^

### Data Analysis

Interviewers took notes and discussed emerging themes as the interviews progressed so that thematic saturation could be assessed. We analyzed the data using constant comparative analysis,^21^ an iterative procedure in which codes and themes evolve as a result of the comparison of new data with previous data. We identified the range of policies and actions considered and/or implemented at participating health care facilities. An initial codebook was used to categorize interventions at the levels of the institution, practitioner, and patient. To manage the data, we used Dedoose qualitative data analysis software, version 8.0.42 (SocioCultural Research Consultants). We focused this article on the range of policies and actions implemented at participating facilities. Data analysis was performed from June 29, 2018, to February 5, 2019.

## Results

We conducted 38 in-depth interviews (26 conducted face to face and 12 conducted by telephone). Interviews spanned 25 institutions, including 13 federally qualified health centers (FQHCs), 7 academic or private hospitals, and 5 public institutions, across the 5 states. Most of the interviews were conducted in California (n = 10), followed by New York (n = 9), Texas (n = 8), Illinois (n = 7), and Florida (n = 4). The interviewees included individuals with clinical and/or administrative positions (n = 27) and senior executives (n = 11).

Although we had originally asked about institution-level, practitioner-level, or patient-level interventions, our analyses found that interviewees described policies and actions that mitigated one or more of the following perceived risks: (1) risk of exposure to immigration enforcement personnel at or near facilities, (2) risk of immigration status–related information disclosure, (3) risk associated with patient-level stressors, (4) risk associated with practitioner-level stressors, and (5) coordination of risk mitigation. The Table lists subcomponents of these categories that emerged from the interviews.

**Table.** Health Care Facility Risk-Reduction Strategies

Category	Policies and actions
Risk of immigration enforcement personnel on or near facilities	Implementing a policy that limits cooperation with immigration enforcement personnel Designating public and private spaces Pursuing alternative models for providing health care services (eg, telehealth)
Risk of immigration status–related information disclosure	Limiting acquisition and documentation of immigration status in medical records Ensuring protection and confidentiality of patient information Offering alternative payment models
Risks associated with patient-level stressors	
Legal stressors	Pursing medical-legal collaborations to meet the legal needs of immigrants Educating patients about their legal rights Incorporating deportation preparedness into larger patient emergency preparedness
Resiliency promotion	Promoting affirming care messages Finding ways to nurture empowerment and engagement (eg, advocacy skills, media and story-telling skill-building programs, and voter registration) among immigrants
Risks associated with practitioner-level stressors	Providing supportive services for employees who are immigrants Educating and offering clinicians health-focused training for providing care to immigrants
Coordination of risk mitigation	Designating an immigration point person or task force

Interviewees at all facilities reported that they addressed at least one of these risk categories. A few facilities had implemented policies or actions that addressed all risk categories, with the exception of 2 FQHCs. Interviewees at most facilities emphasized that their policies and actions fit within a larger mission of addressing the social needs of diverse patients and mitigating perceived risks among patients.

### Immigration Enforcement

Many administrators and employees of health care facilities perceived the presence of immigration enforcement personnel on their premises as a risk to their patients and reported that they had implemented internal protocols regarding the ways in which staff members should respond in such an event. The administrators of the facilities shared several rationales for their internal policies, including the intention to reassure patients and staff members about what the law permits on the premises, prepare them for a worst-case scenario, and reduce fear and its spread in the local community. One FQHC administrator in Texas described the spread of fear “like tuberculosis, a public health issue.”

Interviewees described considering or adopting a range of active and reactive staff protocol components. Examples of these components included requiring visitors to present identification and describe the purpose of their visit upon entry; establishing a code or phone number to alert staff of the presence of immigration enforcement personnel on the premises, thereby activating a facility-wide response system; documenting enforcement officers’ names, badge numbers, and affiliations; documenting enforcement officers’ actions with photographs or video; training an internal team to respond; and determining if and when to notify patients. The personnel at one facility had practiced these protocols as “a drill [because] you never know” and reported that, as part of a training exercise, they had “pulled out a patient and had them be the person that’s being detained.” They informed patients in advance that this was a training simulation to avoid the spread of fear. Other facilities shared the protocols with personnel but not with patients.

Administrators of health care facilities reported preparing their personnel to recognize and distinguish between administrative warrants, judicial warrants, and subpoenas. At most facilities with a training protocol, personnel had focused on preparing a core response team—stating that “our directive was that you should call senior management”—to reduce the burden on health care practitioners who “were terrified this was going to be all on them” in terms of engaging immigration enforcement personnel. Interviewees at a few facilities reported developing a response protocol that was integrated with a larger rapid response team that included attorneys, community leaders, and city officials.

At some health care facilities, staff members delineated differences between public and private spaces on the premises because law enforcement personnel are legally permitted to enter only public, but not private, spaces without a judicial warrant or permission from the facility.^22^ Specific policies included performing an internal review and environmental scan of current signage, posting clear signage in areas requiring further clarity, and sharing information with staff. Interviewees from several facilities noted that the physical layout of the facility constrained their choices. A senior-level executive at an FQHC in California explained that “at our main sites, you can’t get into the waiting area without going through security. In our smaller sites, you walk straight into the waiting area, which is a public space. We should have thought of that.” One facility used a table as a room divider to create a public waiting area for all patients and a second waiting area for patients who had already signed in. A practitioner at an academic medical center in New York expressed concern about people being “afraid because, at a lot of hospitals, they ask for IDs at the front desk,” highlighting the potential for unintended consequences at facilities that had not established community trust.

To address the risk of exposure to immigration enforcement personnel who were en route to a health care facility, staff members at some facilities had established or expanded telemedicine services or offered home visits. Employees at facilities that had expanded such activities reported mixed results. One employee at a California FQHC reported that their telemedicine expansion “failed horribly. A lot of our patients are not technologically savvy. We tried to do the calling… [but] half the time we had the wrong number or [the patients] used a burner phone.”

### Immigration Status–Related Information Disclosure

Interviewees at health care facilities noted that asking about and documenting immigration status could stigmatize patients, discourage them from seeking care, and expose them to unnecessary risk should immigration enforcement officers gain access to medical records. Staff members at some facilities reviewed intake forms and clinical assessment and documentation practices to ensure this information was not being requested or recorded. This policy became formal at some facilities, informal at others, or was practiced only by select health care practitioners at others. One county administrator in Florida described their facility’s policy as a “don’t ask, don’t tell” attitude among practitioners and patients, while another practitioner reported telling patients, “I just need to know the parts where I can help you. I don’t need to know your whole story.” Several interviewees, however, noted that the effort to avoid eliciting patients’ immigration information was counter to a broader effort to assess the social factors associated with patients’ health.

In addition, administrators at several facilities clarified with employees that existing patient privacy laws, such as the Health Information Portability and Accountability Act, applied to immigration status and medical records. Administrators at other facilities reported an emphasis on best practices, such as ensuring that patient information was not in plain view. Some facilities posted information about confidentiality using clear visible signage. One senior executive leader at an FQHC in California expressed her view that patients should be given more information about risks and benefits to make the best-informed decisions, stating, “I can't guarantee anyone their information is safe. How many times has [nearby tertiary academic medical center] been breached? We're small potatoes compared with [them]. I'm never going to promise fake things. They [the patients] have to choose whether they disclose it or not.”

Some health care facilities devised unique payment models, such as out-of-pocket bundled payments for prenatal visits among pregnant individuals who refused to enroll in Medicaid owing to concerns about data sharing with federal agencies, which they feared would have consequences on their future immigration status through public charge policies.

### Patient-Level Stressors

#### Legal Stressors

Risks associated with patient-level legal stressors were addressed in several ways. First, administrators and employees at health care facilities recognized that many patients struggled with daily fears and legal issues associated with their immigration status. In response, some facilities expanded or initiated new medical-legal partnerships focused on providing legal immigration assistance. Other facilities included legal partners in local resource fairs or resource guides, or they coordinated efforts to have practitioners contribute to patients’ legal cases (eg, provide medical evaluations for asylum cases or letters of support to prevent the deportation of family members). One administrator at an FQHC in California explained their facility’s model of offering free legal consultations to patients, stating, “For the patients that we serve, [a legal consultation] can be a fortune. We developed an agreement where particular law firms would be listed in our brochure free of charge. Then, as long as the person said, ‘[clinic] is sending me,’ that consultation would be free.”

Second, personnel at health care facilities addressed confusion and misinformation among patients by providing a Know Your Rights educational program, which is traditionally offered by legal or community-based organizations. These facilities offered brochures, wallet cards, or other informational packets in examination rooms or clinic waiting areas. In a few facilities, clinic personnel or community health workers delivered the Know Your Rights educational program. Employees at these facilities integrated some of their health educational program with immigration educational efforts, saying, “Half the time when the promotoras [community health workers] are out, they give one particular session on cancer education and screening, then follow it up with nutrition and fitness, and then go into immigration.” Some interviewees reported difficulty in providing up-to-date accurate information to patients “because of all the uncertainty right now.” Another practitioner in Texas recommended that her patients speak to an attorney rather than offering advice herself.

Third, personnel at health care facilities also prepared patients for the risk of deportation, which was sometimes incorporated within broader emergency preparedness plans. Deportation preparedness involved “who do you leave your children to, where are their birth certificates, who's going to handle your bank account should you be deported. It becomes a full kit so that everybody knows exactly what to do in the time of emergency.”

#### Promotion of Resiliency

Administrators and staff at some health care facilities promoted resiliency among patients and their families, seeking to respond both reactively and actively to perceived risks. One common active action was the provision of affirming care messages, such as “you are welcome here.” The form of dissemination and the language used to communicate this message varied based on the local facility’s context within the larger community. A county administrator in Florida noted that they had to consider negative feelings about immigration in the local community before taking action, stating, “We don’t want a red target on the organization.”

Most interviewees reported that they avoided using the word sanctuary because it was falsely reassuring, too politically charged, too ambiguous (ie, did not have a definitive meaning), or did not translate well in the languages spoken by their patients. In the words of an administrator at an FQHC in California, “There is a connotation around sanctuary where people believe they can stay there, [that] they can lock themselves in the clinic, be bathed, clothed, and given food and shelter. [But] no. This is a community health center. We cannot do that.” A senior-level executive at an FQHC in Illinois stated, “We can’t promise people something we can’t be. We cannot harbor patients. We cannot hold someone longer than needed for clinical care.” Some interviewees emphasized the importance of providing accurate messages in multiple languages.

Several interviewees reported using patient-empowerment strategies, such as (1) the development of community advisory boards with representation from immigrants; (2) the creation of targeted programming, such as summer youth programs, story-telling events, or media advocacy skill-building programs; (3) outward engagement and policy engagement opportunities, such as visits to local or state legislators; and (4) voter registration efforts to encourage civic participation. One administrator in Texas explained, “We've been taking on multiple initiatives on registering people to vote, empowering them to say this is why it's so important that you go out and vote.”

Interviewees underscored the importance of “partnerships with community-based organizations and legal help organizations [that] are strong” so that “they are eyes and ears on the ground getting constant feedback in both directions.” Interviewees at a number of health care facilities stated that their successes in implementation were owing to the involvement of community stakeholders as active partners, unifying health care facility and community interests regarding the health of immigrants. Community partner organizations varied and included local police forces and foreign consulates. For example, one interviewee discussed a collaboration with the Mexican consulate to provide patients with health education and referrals to case management or primary care.

### Practitioner-Level Stressors

Another identified theme was the consequence of stressors among health care practitioners. Administrators of health care facilities responded by bolstering their legal services and increasing behavioral health support for their personnel. One senior level administrator in Illinois explained, “Many of our staff are from the communities we serve, so this was personal for [them].” Some interviewees at facilities reported hearing biased and demeaning remarks from patients, such as, “Now that Trump's elected, you can't speak Spanish here. They're going to kick you back.”

Interviewees at several facilities emphasized the importance of discussing patients’ anxiety and fears among clinic personnel, addressing sources of burnout, brainstorming ideas, or sharing best practices. These facilities hosted town hall events or dedicated staff meetings to discussing immigration status–related concerns.

Furthermore, interviewees at health care facilities described practitioner education as a crucial component in assisting patients who are immigrants, particularly because practitioners have different levels of experience, training, and comfort in navigating these patients’ concerns. A number of interviewees reported addressing practitioner misconceptions, such as the belief that only non-English speakers are immigrants. Health care facilities that offered education for clinicians focused on reviewing the changes to local and federal government immigration policies; providing guidance for communicating with patients who have immigration status–related questions, including whether and how to ask patients about their immigration status without eliciting fear or stigma; and explaining the ways in which immigration status is associated with health. Interviewees noted that conversations about immigration status were especially delicate, saying, “it’s like you’re in a forest, it hasn’t rained in a year, and anything can just start a fire.” In addition, several senior executive leaders emphasized the limits to clinicians’ knowledge about immigration issues because “they’re doctors, not lawyers, and this is a very changing environment.”

### Coordination of Risk Mitigation

Interviewees at a number of health care facilities reported that, given the pace of change in immigration policy, they had assigned a point person or team designated to stay abreast of policy changes and ensure clinicians and executive leadership were updated about best practices. The process of identifying and connecting with key stakeholders may have otherwise been delayed, which may have hindered the facility’s ability to respond effectively. Team representatives commonly included administrators, frontline practitioners across departments (eg, physicians, nurses, and social workers), and risk management personnel. The point person or team was either newly created or incorporated within existing groups. As one administrator in Illinois explained, “[Our team’s] original purpose was to work…on community outreach. But now, this committee is in charge of reviewing the immigration guide.”

## Discussion

This exploratory multistate qualitative study identified several main areas of response by health care facilities to perceived immigration-associated risks among patients and practitioners, thereby creating an environment that was both physically and psychologically safe. The range of policies and actions described in this study highlights the ways in which health care facilities can implement both active and reactive measures that address risks to the health of immigrants. Previously discussed efforts to improve the health of these patients after the 2016 presidential election have focused on individual patient-practitioner communication^23^ or policy changes at the federal or state level. However, these approaches did not capture the role of health care facilities and the health systems in which they are embedded. The description of these policies and actions is an essential first step in building an evidence base for health system approaches that optimize health care among immigrants. An understanding of these policies and actions may also serve as a guide to personnel at other health care facilities who seek to address the needs of all their patients, regardless of immigration status.

Among most of the facility personnel we interviewed, these policies and actions fit within a larger patient-centered or health equity mission and did not represent controversial actions. Historical and current parallels exist regarding such immigration status–related policies and actions, as health care facilities have responded to nonmedical perceived risks to emphasize their commitment to patients regardless of the political or legal landscape. For example, in war zones, hospitals use distinctive emblems, such as large flags or red crosses on white roofs, to underscore their independent status.^24^ Furthermore, although immigration violations are civil and not criminal, parallels can be drawn with patients with substance use disorders who have committed illegal acts but have continued to have their clinical care prioritized. In addition, personnel at health care facilities have sought to reduce the risks associated with the disclosure of sensitive information in patients’ medical records. This approach was used when HIV infection and AIDS first emerged; during this period, clinicians were hesitant to record the HIV status of patients because of the potential for stigma and discrimination. In the past, practitioners were also reluctant to share patients’ DNA pedigrees and other genetic information that could be used by insurance companies or employers to target asymptomatic patients based on their genetic susceptibilities.^25^

Personnel at health care facilities have also tried to make their environments welcoming, trusting, and supportive by addressing patient and practitioner stressors in other contexts. The connection of patients with legal resources, for example, is akin to the prescription of fruits and vegetables for patients with food insecurity.^26^ Deportation preparedness or planning is similar to advanced care planning. Another example is the fostering of lesbian, gay, bisexual, transgender, and queer (LGBTQ)–friendly health care services.^27^ The Human Rights Campaign, one of the largest civil organizations working to achieve LGBTQ equality, publishes an annual Healthcare Equality Index,^28^ a benchmarking tool that evaluates health care facilities’ policies and practices for the equity and inclusion of patients and employees with LGBTQ identification. Some of the criteria used in the benchmarking tool mirrors the policies and actions that we found had been implemented by health care facilities to serve immigration status–related needs, such as staff training, welcoming signs, and the development of an internal planning or advisory committee focused on issues associated with the care of patients with LGBTQ identification. The creation of age-friendly health systems^29^ to meet the needs of older adults represents the ways in which health care facilities have sought to accommodate patients with less-stigmatized demographic characteristics.

Policies and actions implemented in the immigration context are similar to those that health care facilities have been performing across the disease and policy spectrum for many years, such as reducing risks to their patients, responding to the physical and mental health needs of their patients and employees, and fulfilling their mission for care equity. Coincident with a mission for care equity, these policies and actions were implemented alongside other efforts that addressed common barriers to care, such as language, transportation, or insurance barriers.

### Limitations

Our study had several limitations. The study did not evaluate the policies and actions reported by interviewees. Future studies can assess the efficacy of these interventions and evaluate which variations are most effective. Although this study was not statistically representative, it was purposefully designed to identify a range of perspectives from facilities in regions with large populations of immigrants but with potentially different political pressures and health care delivery challenges. The results suggest that institutions of various sizes and structures across states with different political leanings could potentially apply these policies and actions. Other studies have suggested that the prevalence of fear of deportation is not associated with the use of medical care at FQHCs, in particular,^30^ so the variations across health care facility types require further exploration.

## Conclusions

Population health and immigration policies are at the forefront of current policy debates. An understanding of the ways in which health care facilities serve to mitigate risks to their patients and employees can be one step toward optimizing care for immigrants and health care practitioners. Health care facilities can, in changing policy environments, consider and implement processes to adapt to and address evolving patient and practitioner concerns.

## References

[1] Fleming PJ, Lopez WD, Mesa H, A qualitative study on the impact of the 2016 US election on the health of immigrant families in Southeast Michigan. BMC Public Health. 2019;19(1):. doi:10.1186/s12889-019-7290-3 31307435 PMC6631662

[2] Gemmill A, Catalano R, Casey JA, Association of preterm births among US Latina women with the 2016 presidential election. JAMA Netw Open. 2019;2(7):e197084. doi:10.1001/jamanetworkopen.2019.7084 31322687 PMC6647358

[3] Page KR, Polk S Chilling effect? post-election health care use by undocumented and mixed-status families. N Engl J Med. 2017;376(12):e20. doi:10.1056/NEJMp1700829 28273008

[4] Eskenazi B, Fahey CA, Kogut K, Association of perceived immigration policy vulnerability with mental and physical health among US-born Latino adolescents in California. JAMA Pediatr. 2019;173:744-. doi:10.1001/jamapediatrics.2019.1475 31233132 PMC6593622

[5] Krieger N, Huynh M, Li W, Waterman PD, Van Wye G Severe sociopolitical stressors and preterm births in New York City: 1 September 2015 to 31 August 2017. J Epidemiol Community Health. 2018;72(12):1147-1152. doi:10.1136/jech-2018-211077 30327451 PMC6252370

[6] Toomey RB, Umana-Taylor AJ, Williams DR, Harvey-Mendoza E, Jahromi LB, Updegraff KA Impact of Arizona’s SB 1070 immigration law on utilization of health care and public assistance among Mexican-origin adolescent mothers and their mother figures. Am J Public Health. 2014;104(suppl 1):S28-S34. doi:10.2105/AJPH.2013.301655 24354823 PMC3924594

[7] White K, Yeager VA, Menachemi N, Scarinci IC Impact of Alabama’s immigration law on access to health care among Latina immigrants and children: implications for national reform. Am J Public Health. 2014;104(3):397-405. doi:10.2105/AJPH.2013.301560 24432880 PMC3953801

[8] Rhodes SD, Mann L, Siman FM, The impact of local immigration enforcement policies on the health of immigrant Hispanics/Latinos in the United States. Am J Public Health. 2015;105(2):329-337. doi:10.2105/AJPH.2014.302218 25521886 PMC4318326

[9] Novak NL, Geronimus AT, Martinez-Cardoso AM Change in birth outcomes among infants born to Latina mothers after a major immigration raid. Int J Epidemiol. 2017;46(3):839-849. doi:10.1093/ije/dyw346 28115577 PMC5837605

[10] Lopez WD, Kruger DJ, Delva J, Health implications of an immigration raid: findings from a Latino community in the midwestern United States. J Immigr Minor Health. 2017;19(3):702-708. doi:10.1007/s10903-016-0390-6 27041120 PMC5399061

[11] Vargas ED, Ybarra VD US citizen children of undocumented parents: the link between state immigration policy and the health of Latino children. J Immigr Minor Health. 2017;19(4):913-920. doi:10.1007/s10903-016-0463-6 27435476 PMC5236009

[12] Hatzenbuehler ML, Prins SJ, Flake M, Immigration policies and mental health morbidity among Latinos: a state-level analysis. Soc Sci Med. 2017;174:169-178. doi:10.1016/j.socscimed.2016.11.040 28043019 PMC5258771

[13] Clarke W, Turner K, Guzman L One quarter of Hispanic children in the United States have an unauthorized immigrant parent. National Research Center on Hispanic Children & Families; 2017 Accessed February 4, 2020. https://www.hispanicresearchcenter.org/wp-content/uploads/2019/08/Hispanic-Center-Undocumented-Brief-FINAL-V21.pdf

[14] Vargas ED, Pirog MA Mixed-status families and WIC uptake: the effects of risk of deportation on program use. Soc Sci Q. 2016;97(3):555-572. doi:10.1111/ssqu.12286 27642194 PMC5026318

[15] Vargas ED Immigration enforcement and mixed-status families: the effects of risk of deportation on Medicaid use. Child Youth Serv Rev. 2015;57:83-89. doi:10.1016/j.childyouth.2015.07.009 26435562 PMC4592159

[16] Saadi A, Ahmed S, Katz MH Making a case for sanctuary hospitals. JAMA. 2017;318(21):2079-2080. doi:10.1001/jama.2017.15714 29049516

[17] Pew Research Center Hispanic Trends Project US unauthorized immigrant population estimates by state, 2016. Pew Research Center website. Published February 5, 2019. Accessed September 7, 2019. https://www.pewresearch.org/hispanic/interactives/u-s-unauthorized-immigrants-by-state/

[18] Thomas DR A general inductive approach for analyzing qualitative evaluation data. Am J Eval. 2006;27(2):237-246. doi:10.1177/1098214005283748

[19] Damschroder LJ, Aron DC, Keith RE, Kirsh SR, Alexander JA, Lowery JC Fostering implementation of health services research findings into practice: a consolidated framework for advancing implementation science. Implement Sci. 2009;4:50. doi:10.1186/1748-5908-4-50 19664226 PMC2736161

[20] Morse JM The significance of saturation. Qual Health Res. 1995;5(2):147-149. doi:10.1177/104973239500500201

[21] Glaser BG The constant comparative method of qualitative analysis. Soc Probl. 1965;12(4):436-445. doi:10.2307/798843

[22] American Civil Liberties Union Northern California Know your rights: immigrant safety in public spaces. ACLU Northern California website. Published August 30, 2018. Accessed August 30, 2018. https://www.aclunc.org/our-work/know-your-rights/know-your-rights-immigrant-safety-public-spaces

[23] Kuczewski MG, Mejias-Beck J, Blair A Good sanctuary doctoring for undocumented patients. AMA J Ethics. 2019;21(1):E78-E85. doi:10.1001/amajethics.2019.78 30672423

[24] Hayward-Karlsson J, Jeffery S, Kerr A, Schmidt H; International Committee of the Red Cross Hospitals for War-Wounded: A Practical Guide for Setting Up and Running a Surgical Hospital in an Area of Armed Conflict. International Committee of the Red Cross; 1999.

[25] Kim G, Molina US, Saadi A Should immigration status information be included in a patient’s health record? AMA J Ethics. 2019;21(1):E8-E16. doi:10.1001/amajethics.2019.8 30672413

[26] Saxe-Custack A, LaChance J, Hanna-Attisha M, Ceja T Fruit and vegetable prescriptions for pediatric patients living in Flint, Michigan: a cross-sectional study of food security and dietary patterns at baseline. Nutrients. 2019;11(6):E1423. doi:10.3390/nu11061423 31242555 PMC6627167

[27] Wilkerson JM, Rybicki S, Barber CA, Smolenski DJ Creating a culturally competent clinical environment for LGBT patients. J Gay Lesbian Soc Serv. 2011;23(3):376-394. doi:10.1080/10538720.2011.589254

[28] Human Rights Campaign. Healthcare equality index 2019. Human Rights Campaign Foundation; 2019. Accessed September 9, 2019. https://assets2.hrc.org/files/assets/resources/HEI-2019-FinalReport.pdf

[29] Fulmer T, Mate KS, Berman A The age-friendly health system imperative. J Am Geriatr Soc. 2018;66(1):22-24. doi:10.1111/jgs.15076 28876455

[30] Lopez-Cevallos DF, Lee J, Donlan W Fear of deportation is not associated with medical or dental care use among Mexican-origin farmworkers served by a federally qualified health center–faith-based partnership: an exploratory study. J Immigr Minor Health. 2014;16(4):706-711. doi:10.1007/s10903-013-9845-1 23736963

